# Traceability and distribution of *Neisseria meningitidis* DNA in archived post mortem tissue samples from patients with systemic meningococcal disease

**DOI:** 10.1186/s12907-017-0049-9

**Published:** 2017-08-16

**Authors:** Berit Sletbakk Brusletto, Bernt Christian Hellerud, Else Marit Løberg, Ingeborg Løstegaard Goverud, Åshild Vege, Jens Petter Berg, Petter Brandtzaeg, Reidun Øvstebø

**Affiliations:** 1 Blood Cell Research Group, Section for Research, Department of Medical Biochemistry, Oslo University Hospital HF, Ullevål Hospital, PO Box 4956 Nydalen, 0424 Oslo, Norway; 2 Institute of Clinical Medicine, University of Oslo, Oslo, Norway; 3 Department of Pathology, Oslo University Hospital, Oslo, Norway; 4 Institute of Immunology, Oslo University Hospital, Oslo, Norway; 5 Section for Forensic Pediatric Pathology, Department of Forensic Sciences, Oslo University Hospital, Oslo, Norway

**Keywords:** Systemic meningococcal disease, *Neisseria meningitidis*, FFPE, ctrA gene, Quality of archived tissue

## Abstract

**Background:**

The pathophysiology and outcome of meningococcal septic shock is closely associated with the plasma level of *N. meningitidis* lipopolysaccharides (LPS, endotoxin) and the circulating level of meningococcal DNA. The aim of the present study was to quantify the number of *N. meningitidis* in different formalin-fixed, paraffin-embedded (FFPE) tissue samples and fresh frozen (FF) tissue samples from patients with systemic meningococcal disease (SMD), to explore the distribution of *N. meningitidis* in the body.

**Methods:**

DNA in FFPE and FF tissue samples from heart, lungs, liver, kidneys, spleen and brain from patients with meningococcal shock and controls (lethal pneumococcal infection) stored at variable times, were isolated. The bacterial load of *N. meningitidis* DNA was analyzed using quantitative real-time PCR (qPCR) and primers for the capsule transport A (ctrA) gene (1 copy per *N. meningitidis* DNA). The human beta-hemoglobin (HBB) gene was quantified to evaluate effect of the storage times (2-28 years) and storage method in archived tissue.

**Results:**

*N. meningitidis* DNA was detected in FFPE and FF tissue samples from heart, lung, liver, kidney, and spleen in all patients with severe shock. In FFPE brain, *N. meningitidis* DNA was only detected in the patient with the highest concentration of LPS in the blood at admission to hospital. The highest levels of *N. meningitidis* DNA were found in heart tissue (median value 3.6 × 10^7^ copies *N. meningitidis* DNA/μg human DNA) and lung tissue (median value 3.1 × 10^7^ copies *N. meningitidis* DNA/μg human DNA) in all five patients. *N. meningitidis* DNA was not detectable in any of the tissue samples from two patients with clinical meningitis and the controls (pneumococcal infection). The quantity of HBB declined over time in FFPE tissue stored at room temperature, suggesting degradation of DNA.

**Conclusions:**

High levels of *N. meningitidis* DNA were detected in the different tissue samples from meningococcal shock patients, particularly in the heart and lungs suggesting seeding and major proliferation of meningococci in these organs during the development of shock, probably contributing to the multiple organ failure. The age of archived tissue samples appear to have an impact on the amount of quantifiable *N. meningitidis* DNA.

## Background

Meningococcal infections remain a major public health problem worldwide with a case fatality rate (CFR) of 7-11% in sporadic cases increasing to 20-52% in outbreak situations in Europe and USA [1–3]. Approximately 30% of patients with systemic meningococcal disease (SMD) in Europe develop septic shock [2, 3]. Circulatory collapse is the primary cause of death owing to the combined effect of extreme vasodilation and septic cardiac failure resistant to treatment. The pathophysiology of SMD is closely associated with the ability of *N. meningitidis* to proliferate in the blood and subsequently invade the meninges, as documented by qPCR [2, 4–11]. Fulminant meningococcal septicemia, the most feared clinical presentation, is characterized by rapid progression of septic shock, large petechiae and ecchymoses, disseminated intravascular coagulation (DIC) and renal and pulmonary failure [1, 2, 9, 12, 13]. Post mortem examinations reveal hemorrhagic adrenals in the majority of the patients, “shock”-kidneys often with multiple thrombi in glomeruli, normal or congested lungs sometimes with thrombi and occasionally inflammatory foci in epi- and myocardium [10, 13]. The findings are in line with those described by Ferguson and Chapman [14].

Patients presenting with distinct symptoms of meningitis without shock is the most common presentation and have a much better prognosis than those with shock [1–3]. The number of meningococci and the LPS level in the plasma in these patients are low or undetectable [11] while the levels of meningococcal DNA, LPS and cytokines are 100- to 1000-fold higher in cerebrospinal fluid (CSF) than in blood [1, 2]. Death may be caused by brain edema and herniation of cerebellum.

The pathophysiology and outcome of meningococcal septic shock is closely associated with the plasma level of *N. meningitidis* lipopolysaccharides (LPS, endotoxin) and the circulating level of meningococcal DNA [4, 6, 11]. A rapid proliferation of *N. meningitidis* will result in huge amounts of LPS-containing material in plasma, and 95% of the patients with LPS levels in plasma above 10 endotoxin units (EU) /mL develop persistent shock with a detrimental activation of the innate immune system [1, 2, 9, 10, 12, 15, 16]. LPS, present in the membrane of the bacteria, are the most important but not the only meningococcal molecules that induce inflammation [17, 18].

A few studies have previously addressed detection and distribution of *N. meningitidis* DNA in FFPE human tissue [19, 20] but none has compared the results with FF tissue from the same post mortem examination. Recently, a porcine model of meningococcal septic shock, using the heat inactivated *N. meningitidis*, documented that large numbers of meningococci accumulated in the lungs, heart, liver, spleen and kidneys inducing a massive organ inflammation [21]. Similar results were found in three patients with lethal meningococcal septic shock by examining fresh frozen (FF) tissue from the same organs [21]. As an extension of this study, we aimed to detect and quantify *N. meningitidis* DNA i.e. copy numbers using qPCR in tissue samples from different organs obtained by post mortem examination. The tissue samples were formalin-fixed, paraffin-embedded (FFPE) and stored at room temperature (20 – 25 °C) for up to 28 years. A human endogenous DNA control was included to evaluate the effect of storage time on degradation of tissue samples. Furthermore, we compared the qPCR results obtained from FFPE tissue stored at 20 – 25 °C with FF tissue stored at −80 °C for up to six years.

## Methods

### Clinical definitions

Systemic meningococcal disease (SMD) was present if *N.meningitidis* was cultivated or (−and) confirmed by polymerase chain reaction (PCR) in blood and/or (CSF) [4, 11].

Severe septic shock was defined as persistent hypotension because of bacterial infection, with an initial systolic blood pressure < 90 mmHg in adults (≥ 12 year) and <70 mmHg in children (< 12 year), that required fluid therapy and treatment with vasoactive drugs (dopamine, epinephrine, norepinephrine) for at least 24 h or until death [4].

Transient shock was defined as hypotension, as defined above, requiring volume treatment combined with vasoactive drugs for less than 6 h to stabilize the circulation.

Multiple organ failure was defined as: 1) reduced pulmonary function requiring artificial ventilation to maintain an adequate arterial oxygenation and 2) renal failure with reduced creatinine clearance (<60 mL /minute per 1.73 m^2^ body surface) or pathologically elevated serum creatinine (related to age and collected within 12 h after admission).

Clinical meningitis was defined as nuchal and back rigidity with positive Kernig’s and/or Brudzinski’s signs and pleocytosis with ≥100 × 10^6^ leukocytes/L CSF.

### Subjects

Altogether seven patients were included in the study. Five patients (No 1 – 5) had severe shock without clinical meningitis whereas patient 6 had clinical meningitis without shock and patient 7 had clinical meningitis and transient shock (Table 1).

**Table 1:** Patients with systemic meningococcal disease and their clinical characteristics

Patient No Serogroup	Neisserial DNA; copy number of *N.meningitidis/*mL LPS (LAL); EU/mL at admission to hospital* not available	Age of tissue at isolation time of DNA (years)	Type of storage methods	Type of organ tissue	Findings at autopsy # no obviously pathological changes
1 Serogroup B	2.8x10^8^copies/mL (plasma) 2100 EU/mL (plasma) No spinal puncture was performed	11	FFPE	Skin Adrenal glands Lungs Heart Liver Kidneys Spleen Brain	Skin hemorrhages Hemorrhage Edema Petechiae on epicard and endocard # Dark-red congested medulla and pale cortex (shock kidneys). Fibrin thrombi in glomeruli. # Fibrin thrombi in vessels in choroid plexus
2 Serogroup B	3.8x10^7^copies/mL (plasma) 271 EU/mL (plasma) No spinal puncture was performed	10	FFPE	Skin Adrenal glands Lungs Heart Liver Kidneys Spleen Brain	Skin hemorrhages Hemorrhage An localized area with atelectasis, some neutrophils and small hemorrhages. Fibrin thrombi in vessels. Petechiae on epicard # Congested vessels in medulla and fibrin thrombi in glomeruli # Fibrin thrombi in some vessels
3 Serogroup B most likely	1.0x10^8^copies/mL (serum) 2140 EU/mL (serum) Spinal puncture was performed post mortem. CSF contained 8 EU/mL	5 5	FFPE FF	Skin Adrenal glands Lungs Heart Liver Kidneys Spleen Brain	Skin hemorrhages Hemorrhage # Petechiae on epicard # Dark-red congested medulla and pale cortex (shock kidneys) # #
4 Serogroup C	3.0x10^7^copies/mL (serum) 3800 EU/mL (serum) No spinal puncture was performed	2 2	FFPE FF	Skin Adrenal glands Lungs Heart Liver Kidneys Spleen Brain	Skin hemorrhages Hemorrhage Congestion Petechiae on epicard. Microabscess in myocard # Congested vessels in medulla and multiple fibrin thrombi in glomeruli # #
5 Serogroup C	* * *	6 6	FFPE FF	Skin Adrenal glands Lungs Heart Liver Kidneys Spleen Brain	Skin hemorrhages Hemorrhage # # # # # #
6 Serogroup B	0.25 EU/mL (plasma) *	28	FFPE	Lungs Heart Liver Kidneys Spleen Brain	Edema # # # # Edema, herniation of cerebellum, pus in meninges
7 Serogroup B	1.1x10^5^copies/mL (plasma) 2.1 EU/mL (plasma) 4000 EU/mL (CSF)	28	FFPE	Lungs Kidneys Brain	Edema # Edema, herniation of cerebellum, pus in meninges

*Formalin-fixed, paraffin-embedded (FFPE) tissue from patients with meningococcal shock and multiple organ failure (patient No 1 – 5):* The formalin-fixed, paraffin-embedded tissues were selected according to histopathological findings; presence of neutrophilic inflammatory infiltrates or thrombi. Small tissue specimens from five lungs, four hearts, four livers, four kidneys, three spleens and four brains were available.

The samples were collected during the routine post mortem examination within 24 h after the patient died. The storage times of the FFPE tissue samples were 11, 10, 6, 5 and 2 years (Table 1).

*Fresh frozen (FF) tissue specimens from patients with meningococcal shock and multiple organ failure (patient No 3 – 5):* Three lungs, three hearts, two livers, three kidneys, three spleens and one brain were collected in parallel with the routine post mortem examination and frozen at −80 °C for later analysis. The storage times of the FF tissue were 6, 5 and 2 years. The samples had been partially thawed once and examined before this analysis [21].

*Formalin-fixed, paraffin-embedded (FFPE) tissue from patients with clinical meningitis and herniation of cerebellum (No 6 and 7):* FFPE tissue from both patients were stored for 28 years. FFPE tissue samples from lungs, liver, spleen, kidneys and brain from patient No 6 and lung, kidney and brain from patient No 7 were analyzed.

*Formalin-fixed, paraffin-embedded (FFPE) tissue from patients with lethal systemic pneumococcal infection (negative controls):* FFPE tissue from two patients with microbiologically verified lethal pneumococcal infection with positive blood cultures served as negative controls. The storage time of the specimens was four and six years, respectively. Tissue from lungs, heart, liver, spleen, kidneys and brain were analyzed. The organ samples were collected at routine post mortem examination 24-48 h after death.

### Autopsy procedure

The study was carried out at the Department of Pathology, Oslo University Hospital, Department of Pathology Stavanger University Hospital, and at the Section for Forensic Pediatric Pathology, Oslo University Hospital, Oslo, Norway (former: Department of Research and Development in Forensic Pathology, The Norwegian Institute of Public Health, Oslo, Norway). All the autopsies have been carried out by pathologists on duty and according to routine procedures (which include sterile equipment for microbiological sampling).

### Fixation and paraffination procedure

All FFPE tissue samples were prepared according to routine procedures. The protocol for fixation of most tissue used in this study was as follows: The tissue samples were fixed in 4% buffered –neutral formalin, at room temperature for 6-48 h. Thereafter the tissue blocks were dehydrated, cleared and infiltrated with the embedding material in automated tissue processors ready for external embedding in paraffin.

The whole brain was fixed in unbuffered formalin for at least 3 weeks at room temperature before samples from different regions of the brain were processed further in an automated tissue processor.

All tissue samples stored for 28 years before DNA isolating time (meningitis patients) had been fixed in unbuffered formalin, at room temperature for 6-24 h. Thereafter the tissue blocks were prepared as the other tissue samples.

### Tissue staining

Tissue sections, 3 μm thick, were placed on slides, deparaffinized, and rehydrated through degraded alcohols and distilled water. Then the sections were stained with hematoxylin and eosin (HE) staining and Acid Fuchsin Orange K (AFOG) staining. All microscopy slides were examined by an experienced pathologist.

### DNA extraction

DNA from freshly cut slices of five 10 μm-thick sections of archival FFPE blocks was isolated in parallel samples. The samples with the sections were immediately placed in 1 mL of Xylenes (cat.no: 534,056 Sigma –Aldrich) in a microcentrifuge tube for deparaffinization. The QIAamp DNA FFPE tissue kit (Qiagen, Hilden, Germany) was used for the extraction of DNA in the QIAcube robot www.qiagen.com/MyQIAcube according to manufacturer’s instructions. RNase A was added to degrade RNA in the DNA samples. The DNA was eluted in 40 μL ATE buffer. Negative control (one sample without tissue sample) was subjected for isolation to check for contaminations. DNA samples were stored at −80 °C before further analysis.

DNA isolated in parallel from freshly cut sections of FFPE tissue gave almost similar yield and purity (260/280 ratio) for the parallel sections (data not shown). Therefore, only one of the samples from the parallel isolation of DNA from each tissue was used in the qPCR.

DNA from FF: 50 mg of frozen tissue was placed in 400 μL MagNA Pure DNA tissue lysis buffer (Roche Applies Science, Indianapolis, IN), then homogenized using a Xiril Dispomix (AH diagnostics, Aarhus, Denmark). Furthermore the samples were incubated for 30 min at room temperature for lysis, transferred to Nunc tubes and stored at −80 °C until analysis. The MagNA Pure LC DNA Isolation Kit II (Tissue kit) (Roche Applies Science, Indianapolis, IN) was used for extraction of DNA in the MagNA Pure LC Robot (Roche Applies Science, Indianapolis, IN) according to manufacturer’s instructions. The DNA was eluted in 200 μL Elution Buffer and stored at -80 °C before further analysis. DNA concentration and purity (260/280 ratio) were determined with the NanoDrop ND-1000 Spectrophotometer (Thermo Fisher Scientific, Waltham, MA).

### Quantitative real-time PCR

Quantitative real-time PCR (qPCR) with oligonucleotide primers for capsule transport A (ctrA) [22] (GenBank sequence M80593) [7], was used to quantify meningococcal DNA. A standard curve (range 10 ng to 0.01 pg, 7 standards) generated from known amount of DNA isolated from *N.meningitidis* was used for quantification. Each standard point was analyzed in triplicates and a new standard curve was included in every PCR analysis. The PCR efficiency and R^2^ value were 92% and 0.996 (*n* = 13) respectively.

*Sensitivity:* The lower limit of detection (LLD) of the ctrA qPCR assay was 0.01 pg of genomic DNA. The coefficient of inter assay variation (CV %) based on an in house control, (DNA from *N.meningitidis*) was 2.4% measured as obtained cycle threshold (Ct) value and 31.9% when calculated from the standard curve. If no increase in the fluorescence signal was observed after 35 cycles, the sample was assumed to be negative. To rule out false negative results (due to inhibition) all samples were diluted from 100 to 0.01 ng DNA in the qPCR reaction.

*Specificity:* There were no cross-reaction using ctrA primers with human genomic DNA from negative controls, FFPE from patients with lethal pneumococcal infection or from patients suffering from a non-inflammatory disease (data not shown). A negative sample for ctrA had to simultaneously have a Ct value ≤35 for HBB. If not, the DNA in the sample was assumed to be too degraded to be included in the study.

#### Quality of isolated DNA from FFPE and FF tissue

**Fig. 1:**
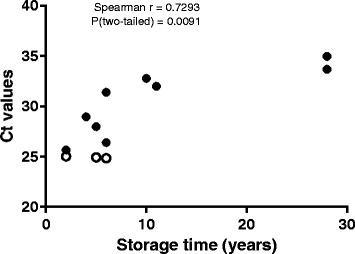
The association between increasing storage time and Ct values of endogenous control HBB in lung tissue from FFPE and FF tissue samples. y-axis show Ct values for HBB gene in lung tissue and x-axis the age of lung tissue at DNA isolation time. The association between increasing storage time and Ct shown as r (spearman) = 0.73, *p* = 0.009. Empty circles are FF tissue samples. Filled circles represent FFPE tissue samples

To evaluate the quality of the DNA extraction and to verify the presence of amplifiable DNA, amplification of the human beta-hemoglobin (Human hemoglobin, beta HBB Hs00758889_s1, Thermo Fisher TaqMan® gene expression assay) [23–25] from every patient sample was analyzed. The inter assay variation (CV %) of an in house endogenous HBB control was 2.9% (Ct values). The association between storage time and Ct values was evaluated by quantification of HBB in lung tissue samples from FFPE and FF (Fig 1).

#### Quality of DNA to quantitative real-time PCR

To check for inhibition in the PCR reaction and to determine amount of input DNA, serial dilutions (ranging 100-0.1 ng) of DNA was used [20, 26]. DNA (5 μL) was amplified in 25 μL reaction volumes containing (Life technologies) 1.25 μL TaqMan® Gene expression assay (20X), 12.5 μL TaqMan® Universal Master Mix II (2X) and 6.25 μL RNase-free water.

Input of 100 ng DNA to the PCR reaction (quantified by the NanoDrop ND-1000 Spectrophotometer) was found appropriate for both *N. meningitidis* DNA and HBB quantification. The assays were carried out with the ViiA™ 7 Real-Time PCR systems (Applied Biosystems by Life Technologies, Carlsbad, CA 92008 USA) using the following cycling parameters: 50 °C for 2 min, then 95 °C for 10 min followed by 40 cycles of a 2-stage temperature profile of 95 °C for 15 s and 60 °C for 1 min. If no increase in the fluorescence signal was observed after 35 cycles, the sample was assumed to be negative. The final calculation of the bacterial load (copies *N. meningitidis* DNA/μg tissue DNA) and HBB gene (Ct) from samples and controls was performed by the software provided with the Applied Biosystems ViiA™ 7.

Only duplicate positive results for quantification of parallel samples from both *N. meningitidis* DNA and HBB were finally considered as positive. A no template control, a positive control for *N. meningitidis* DNA and a positive control for HBB were included in every run.

### Quantification of *N.meningitidis* DNA and LPS in plasma/serum/CSF from patients with meningococcal disease in samples collected on hospital admission

The heparin-blood was collected, centrifuged, plasma pipetted off and aliquoted as described in detail earlier [4, 27]. Quantification of *N. meningitidis* DNA was performed as previously described in detail [11, 28]. The detection limit was 10^3^ *N.meningitidis* DNA copies/mL.

Quantification of LPS in plasma/serum/CSF was initially performed with an in house developed limulus amebocyte lysate (LAL) assay and later with Chromo-LAL (Associates of Cape Cod, USA) with a detection limit of 0.2 EU/mL. The serum level is on average 63% of the plasma level [4, 27].

### Statistical analysis

The GraphPad Prism Software Version 6.07 (GraphPad Software, San Diego, CA, USA) was used for all statistical analysis.

## Results

### DNA extraction: Yield and purity

DNA isolated from one to six different FFPE tissue samples from five patients with meningococcal shock disease ranged from 33.5 - 282.7 ng/μL, (*n* = 25 and median ng/μL = 83) in concentration and from 1.72-2.05 (260/280 ratio) in purity.

DNA isolated from FFPE tissue samples from patients with meningococcal meningitis ranged from 28.1-90.2 ng/ μL, (*n* = 11 and median ng/μL = 60.3) in concentration and from 1.64-2.16 (260/280 ratio) in purity.

The DNA isolated from FFPE tissue samples from negative controls (*pneumococcal infection)* ranged from 48.3-214.3 ng/μL, (*n* = 12 and median ng/μL = 85.4) in concentration and from 1.38-1.94 (260/280 ratio) in purity.

DNA isolated from FF tissue from three patients with meningococcal disease ranged from 29.5-171.5 ng/μL, (*n* = 15 and median ng/μL = 74.3) in concentration and from1.73-1.98 (260/280 ratio) in purity.

### Evaluation of the quality of isolated DNA from FFPE and FF tissue

The endogenous control HBB quantified in 100 ng FFPE and FF tissue showed variable Ct values dependent on age of storage and storage methods. In FF tissue we found Ct values around 25 (mean Ct value in lung tissue), range 24.84-25.03. In 28 years old FFPE tissue, Ct value was around 34.3 (mean Ct value in lung tissue), range 33.67-34.96. Storage time of tissue sample at DNA isolating time point was positively correlated to HBB Ct values, (Spearman *r* = 0.73, *p* = 0.009, *n* = 12) (Fig. 1).

### Quantification of *N.meningitidis* DNA and LPS in plasma/serum or CSF from patients with severe shock and multiple organ failure

The number of *N.meningitidis*/mL in the circulation of the patients with meningococcal shock ranged from 3.0 × 10^7^/mL to 2.8 × 10^8^/mL (Table 1). LPS in plasma or serum ranged from 271 EU/mL to 3800 EU/mL (Table 1).

Patient 6 with clinical meningitis had LPS values in serum <0.25 EU/mL. CSF was not available for analysis. Patient 7 had *N. meningitidis* DNA 1.1x10^5^copies/mL and LPS 2.1 EU/mL in plasma and 4000 EU/mL of LPS in CSF. CSF for *N. meningitidis* DNA quantification was not available (Table 1).

### Quantification of *Neisseria meningitidis* DNA in FFPE and FF tissue from patients with meningococcal shock and multiple organ failure

**Fig. 2:**
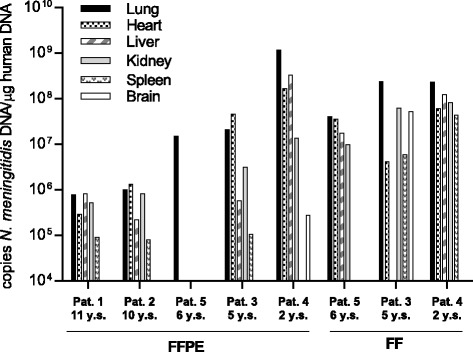
Quantification of *N.meningitidis* DNA in FFPE and FF tissue from patients with systemic meningococcal disease. Input of 100 ng DNA from different tissue samples were quantified using specific *N.meningitidis* primers (capsule transport A) and quantitative PCR. The y-axis show the *N.meningitidis* DNA concentration, the x-axis show patients number (No 1-5), storage methods (FFPE and FF) and storage time at DNA isolating time (y.s)

*N.meningitidis* DNA was detected in FFPE tissue in all patients with severe shock and multiple organ failure (Table 2 and Fig 2). The amount of *N. meningitidis* DNA found in FFPE tissue from each patient showed large variability. For patient 1 and 2 the concentrations of *N. meningitidis* DNA in the organs ranged from 8,1 × 10^4^ -1,3x10^6^copies *N. meningitidis* DNA /μg human DNA. The storage time of these FFPE tissue was above 10 years. In patient 3, 4 and 5 with storage time of five, two and six years, the concentration of *N. meningitidis* DNA was above 1.3 × 10^6^ copies *N. meningitidis* DNA /μg human DNA for most tissue, ranged from1.1 × 10^5^ -1.2 × 10^9^ copies *N. meningitidis* DNA /μg human DNA (Table 2 and Fig 2).

**Table 2:** Quantification of *N. meningitidis* DNA in FFPE and FF tissue from patients with systemic meningococcal disease

Patient No	Age of tissue at isolation time for DNA (years)	Type of organ	Copies *N. meningitidis* DNA/μg human DNA * not available	
			FFPE	FF
1	11	Lung	7.9x10e5	
		Heart	2.9x10e5	
		Liver	8.2x10e5	
		Kidney	5.3x10e5	
		Spleen	9.1x10e4	
		Brain	0	
2	10	Lung	1.0x10e6	
		Heart	1.3x10e6	
		Liver	2.2x10e5	
		Kidney	8.3x10e5	
		Spleen	8.1x10e4	
		Brain	0	
3	5	Lung	2.1x10e7	2.4x10e8
		Heart	4.6x10e7	4.2x10e6
		Liver	5.8x10e5	*
		Kidney	3.2x10e6	6.3x10e7
		Spleen	1.1x10e5	5.9x10e6
		Brain	0	5.2x10e7
4	2	Lung	1.2x10e9	2.3x10e8
		Heart	1.7x10e8	6.1x10e7
		Liver	3.3x10e8	1.2x10e8
		Kidney	1.4x10e7	8.3x10e7
		Spleen	*	4.3x10e7
		Brain	2.8x10e5	*
5	6	Lung	1.5x10e7	4.1x10e7
		Heart	*	3.5x10e7
		Liver	*	1.7x10e7
		Kidney	*	9.9x10e6
		Spleen	*	*
6	28	Lung	0	
		Heart	*	
		Liver	0	
		Kidney	0	
		Spleen	0	
		Brain	0	
7	28	Lung	0	
		Kidney	0	
		Brain	0

Patient 4 had the highest concentration of *N. meningitidis* DNA in all organs of the five patients with severe shock and multiple organ failure, where the concentration ranged from 2.8 × 10^5^-1.2 ×10^9^copies *N. meningitidis* DNA /μg human DNA. In this patient *N. meningitidis* DNA was also detected in the brain tissue with 2.8 × 10^5^ copies *N. meningitidis* DNA /μg human DNA. Lung and liver tissue contained the highest concentration of *N. meningitidis* DNA as compared to the other organs. The storage time of these FFPE samples was only 2 years. This patient also had the highest concentration of LPS in the serum samples collected on hospital admission.

To evaluate the storage methods, we compared the levels of *N. meningitidis* DNA in FF tissue with FFPE tissue from three patients (3, 4 and 5) (Table 2 and Fig. 2). *N. meningitidis* DNA was present in all FF tissue. From patient 3, FF tissue from the brain was available and 5.2x10e^7^ copies of *N. meningitidis* DNA /μg human DNA was detected. In general, we found higher levels of *N. meningitidis* DNA in FF tissue in patient 3 and 5 as compared with FFPE tissue. In patient 4, the amount of *N. meningitidis* DNA was higher in most FFPE tissue compared to FF tissue. The storage time of these samples was only 2 years.

The FF tissue results in patient 4 revealed the highest concentration of *N. meningitidis* DNA in lungs, liver and heart and were in accordance with the results obtained with FFPE tissue. The *N. meningitidis* DNA concentration in FFPE lung tissue however, was four times higher than in FF lung tissue (Table 2).

### Quantification of *Neisseria meningitidis* DNA in FFPE tissue from patients with meningococcal meningitis and cerebellar herniation

*N.meningitidis* DNA was not detected in any FFPE tissue samples, including brain tissue, from the two patients with meningococcal meningitis (storage time 28 years) (Table 2).

### Quantification of *Neisseria meningitidis* DNA in FFPE tissue from patients with lethal pneumococcal infection

*N.meningitidis* DNA was not detected in any of the tissue.

## Discussion

In this study, we detected *N. meningitidis* DNA in FFPE and FF tissue from the lungs, heart, liver, kidneys and spleen in five patients dying of severe meningococcal shock and multiple organ failure. The highest concentration of *N meningitidis* DNA was found in the lungs and hearts. In one patient (No 4), with the highest levels of meningococcal DNA and LPS in plasma, *N. meningitidis* DNA was also detected in the brain FFPE tissue. In FF brain tissue from patient No 3 *N. meningitidis* DNA was also detected. Our results are in line with the current view that fulminant meningococcal septic shock and multiple organ failure is a compartmentalized infection with a massive proliferation of the bacteria in the circulation spreading to different large organs and the skin where they accumulate in large number. *N. meningitidis* can be detected in the CSF in shock patients but usually in much lower number as compared with patients with clinical symptoms of meningitis [4, 5, 11]. Our results are also in line with Fernández-Rodríguez et al. and Guarner et al. who detected *N. meningitidis* DNA in 81-100% of FFPE tissue from patients with meningococcal sudden deaths [19, 20]. Our previously published results also suggest that the majority of the nonviable bacteria could be disintegrated in various organs still exerting a powerful stimulus of the local innate immune system [21].

Brain tissue from two patients (6 and 7) dying of meningitis resulting in brain edema and cerebellar herniation, were without detectable *N. meningitidis* DNA in the brain and other organs. We know from previous studies that patients with meningitis as a group have high levels of LPS and inflammatory mediators as well as *N. meningitidis* DNA in CSF [1, 2, 5, 11, 29, 30]. In this study, the LPS level in CSF in one of two meningitis patients (No 7) was 4000 EU/mL, documenting a massive proliferation of meningococci in CSF. The storage time of tissue from brain and other organs, however, was 28 years at room temperature for both meningitis patients. We interpret our negative results in the brain tissue as a consequence of gradual degradation of DNA due to storage time (over 28 years) at room temperature and fixation in unbuffered formalin [31, 32]. Quantitative measurements of HBB DNA suggest that human DNA is degraded over time as indicated in Fig. 1. We assume that the same is the case for bacterial DNA in human tissue stored at room temperature.

In the two patients (No 6 and 7) dying of cerebellar herniation all extracranial organs examined, were *N. meningitidis* PCR negative. In addition to degradation of DNA as discussed above, the negative results could reflect low seeding of the different organs. Both patients had low levels of LPS in plasma (Table 1) indicating a low bacterial load in the circulation and possibly tissue concentrations of *N. meningitidis* below detection level (true negative results) [2, 5, 11]. Since few patients with distinct clinical meningococcal meningitis without shock die, we did not have any recent post mortem tissues to verify this hypothesis.

The patients with lethal pneumococcal disease serving as negative controls, had as expected no detectable *N. meningitidis* DNA. We did not quantify the amount of *Streptococcus pneumoniae* DNA in the different tissue in these two control patients, however, in a recently published study, comprising 11 patients with systemic pneumococcal infections, FF tissue from the large organs contained 10^4^ – 2 × 10^6^ pneumococci per gram tissue, the highest levels detected in the lungs [21].

Does the copy number of *N. meningitidis* in the different tissues represent bacterial components located primarily in the capillaries of the different organs or do they indicate meningococcal molecules, primarily LPS, which trigger tissue macrophages and induce local inflammation? A histological case study of one patient with lethal meningococcal sepsis suggested that meningococci mainly adhered to capillaries located in low flow regions in the infected organs [33]. Presumably they proliferate in the capillary vascular bed [33]. The capillary density is known to vary anatomically and functionally in different organs as well as within a single organ [34, 35]. How capillary density and volume may influence the distribution of *N. meningitidis* in different organs is not investigated in this study. However, we have previously found massive organ inflammation by using multiplex assay and quantified tumor necrosis factor (TNF), interleukin (IL)-1β, IL-6 and IL-8 in homogenized fresh frozen tissue samples from lungs, heart, liver, spleen, kidneys and adrenals from patients 3,4 and 5 [21]. These observations suggest that LPS and other neisserial molecules are recognized by tissue macrophages in the different organs, implying that the bacterial components are located in the tissues and trigger a local immune response varying from organ to organ.

Pre-analytical factors that may have an influence on the DNA analysis of FFPE tissue can be: postmortem interval (PMI), cold ischemia time (time between biospecimen removal from the body and its preservation), specimen size, fixative buffer (unbuffered formalin or neutral buffered formalin), fixative delivery method, fixative temperature and duration, block storage, section thickness and section storage, and methods of nucleic acids isolation [36–41]. Several reports have shown that the FFPE method will lead to degradation of DNA over time due to the formalin fixation of tissue before paraffin embedding [38].

To monitor the quality of DNA extraction and to verify the presence of amplifiable DNA in our study, an endogenous DNA control gene HBB was quantified in FFPE and FF tissue (Fig1) [23, 42, 43]. Not surprisingly, the highest amount of HBB was found in the FF tissue, with Ct values of about 25 which indicates high amounts of high quality DNA as starting point for PCR amplification. When quantifying HBB in FFPE tissue, increasing Ct values, i.e. lower levels of intact DNA, were found with increasing storage time (storage time 28 years showed Ct values around 34) indicating degradation of DNA over time. In accordance with other studies this verifies that storage time of the FFPE tissue block has a major impact on the concentration of DNA found in the tissue [20, 31, 41, 44, 45]. Several reports show that amplification of DNA despite degradation of DNA may be possible [31, 41, 46]. The PCR products that are amplified in this study have amplicons under 100 base pairs. It is highly recommended to design the amplicons to be as short as possible when carrying out the qPCR of DNA from FFPE [47].

Storage time of FFPE tissue and deposit temperature are two parameters that may influence the traceability of specific DNA [20, 31, 41, 44, 45]. We conclude that meningococcal DNA can be detected in different tissue with the present qPCR assay after storage at room temperature for 11 years in meningococcal patients with shock and multiple organ failure. Storage for 28 years at room temperature gave negative results also in brain tissue with expected high concentration of meningococci in CSF on hospital admission. These results are supported by finding a significant positive association (*r* = 0.73) between storage time of tissue and amount of amplifiable endogenous control (HBB). Detectable levels of HBB DNA decline over the years, and suggest a degradation over time. The results are also in line with the findings of Fernández-Rodríguez et al. [20].

To evaluate the storage methods, we compared the levels of *N. meningitidis* DNA in fresh frozen (FF) tissue with FFPE tissue from three patients (3, 4 and 5) (Table 2 and Fig. 2). The tissue were 5, 2 and 6 years old, respectively. *N. meningitidis* DNA was detected in all tissue. In patient 3 and 5, the results showed a greater amount of *N. meningitidis* DNA in most FF tissue samples compared to FFPE, which is in line with previous studies [48, 49]. Patient 4 showed more similar levels of *N. meningitidis* DNA in the FFPE tissue and in FF tissue for most of the samples compared with patient 3 and 5. An explanation might be that the patient samples were only 2 years old and in good quality with less degradation of DNA.

## Conclusion

Important observations from this study are that detection of *N.meningitidis* DNA by qPCR is possible in both FF tissue and in the conventional FFPE tissue. However, fixation methods and storage times are issues that may affect the results. Inclusion of an endogenous DNA control in the assays will give trustworthy results.

This study suggests that N*. meningitidis* DNA is present in high concentrations in many of the major organs in meningococcal patients with shock and multiple organ failure [21]. *N. meningitidis* may induce a strong molecular inflammatory response in various tissues without distinct visible microscopical changes owing to the short duration of the infection before death [21].

## Abbreviations

AFOG: Acid Fuchsin Orange K
CFR: Case fatality rate
CSF: Cerebrospinal fluid
Ct: Cycle threshold
CV: Coefficient of variation
DIC: Disseminated intravascular coagulation
EU: Endotoxin unit
FF: Fresh frozen
FFPE: Formalin-fixed, paraffin-embedded
HBB: Human beta-hemoglobin
HE: Hematoxylin and eosin
IL: Interleukin
LAL: Limulus amebocyte lysate
LLD: Lower limit of detection
LPS: Lipopolysaccharides
PCR: Polymerase chain reaction
PMI: Postmortem interval
qPCR: Quantitative real-time PCR
SMD: Systemic meningococcal disease
TNF: Tumor necrosis factor
